# The innovation of AI-based software in oral diseases: clinical-histopathological correlation diagnostic accuracy primary study

**DOI:** 10.1186/s12903-024-04347-x

**Published:** 2024-05-22

**Authors:** Shaimaa O. Zayed, Rawan Y.M. Abd-Rabou, Gomana M. Abdelhameed, Youssef Abdelhamid, Khalid Khairy, Bassam A. Abulnoor, Shereen Hafez Ibrahim, Heba Khaled

**Affiliations:** 1 Department of Oral maxillofacial Pathology, Faculty of Dentistry, Cairo University, Cairo, Egypt; 2 Department of Oral Pathology, Misr University for Science and Technology, P. O. Box 77, Giza, Egypt; 3 Faculty of Oral Medicine & Dental Surgery, Misr University for Science and Technology, P. O. Box 77, Giza, Egypt; 4 General Medicine, New Giza, Giza, Egypt; 5 Philosophy & Interactive Media Minors, New York University, Abu Dhabi, United Arab Emirates; 6 Kafrel-Shiekh University, Kafrel-Shiekh, Egypt; 7 Fixes Prosthodontics, Faculty of Dentistry, Ain Shams University, Cairo, Egypt; 8 Faculty of Dentistry, Cairo University, Cairo, Egypt; 9 Lecturer of Oral Maxillofacial Pathology, Faculty of Dentistry, Cairo University, Cairo, Egypt

**Keywords:** Artificial intelligence, Oral diseases, Deep learning

## Abstract

**Background:**

Machine learning (ML) through artificial intelligence (AI) could provide clinicians and oral pathologists to advance diagnostic problems in the field of potentially malignant lesions, oral cancer, periodontal diseases, salivary gland disease, oral infections, immune-mediated disease, and others. AI can detect micro-features beyond human eyes and provide solution in critical diagnostic cases.

**Objective:**

The objective of this study was developing a software with all needed feeding data to act as AI-based program to diagnose oral diseases. So our research question was: *Can we develop a Computer-Aided Software for accurate diagnosis of oral diseases based on clinical and histopathological data inputs?*

**Method:**

The study sample included clinical images, patient symptoms, radiographic images, histopathological images and texts for the oral diseases of interest in the current study (premalignant lesions, oral cancer, salivary gland neoplasms, immune mediated oral mucosal lesions, oral reactive lesions) total oral diseases enrolled in this study was 28 diseases retrieved from the archives of oral maxillofacial pathology department. Total 11,200 texts and 3000 images (2800 images were used for training data to the program and 100 images were used as test data to the program and 100 cases for calculating accuracy, sensitivity& specificity).

**Results:**

The correct diagnosis rates for group 1 (software users), group 2 (microscopic users) and group 3 (hybrid) were 87%, 90.6, 95% respectively. The reliability for inter-observer value was done by calculating Cronbach’s alpha and interclass correlation coefficient. The test revealed for group 1, 2 and 3 the following values respectively 0.934, 0.712 & 0.703. All groups showed acceptable reliability especially for *Diagnosis Oral Diseases Software (DODS)* that revealed higher reliability value than other groups. However, The accuracy, sensitivity & specificity of this software was lower than those of oral pathologists (master’s degree).

**Conclusion:**

The correct diagnosis rate of DODS was comparable to oral pathologists using standard microscopic examination. The DODS program could be utilized as diagnostic guidance tool with high reliability & accuracy.

## Introduction

Nowadays, there is a growing interest in the medical field towards artificial intelligence (AI). Interobserver variation in oral pathology diagnosis is a well-recognized in the routine practice. Unfortunately, many errors appear to be the result of inexperienced pathologists oversight clinical signs and symptoms, missing a pathologic finding on a slide or insufficient biopsies [1]. Hence, inaccurate diagnosis inevitably results in inappropriate patient management. The promising advancement in the field of artificial intelligence and machine learning raised hope for reducing human errors and providing more standardized and objective results [2]. 

AI- based mechanisms permit accomplishments of tasks like humans. It utilizes a neural network, mimicking the neurons in the human brain. It requires providing a substantial amount of feeding data, so the algorithm execute gradual recognition and extraction of the hallmarks of the facts, leading to automated evolution of a model through program training [3]. 

Machine learning (ML) through artificial intelligence (AI) could provide clinicians and oral pathologists to advance diagnostic problems in the field of potentially malignant lesions, oral cancer, periodontal diseases, salivary gland disease, oral infectious lesions, oral immune mediated disease, and others. AI could provide numerous opportunities in critical diagnosis and deal with challenges that face clinicians as it detects micro-features beyond human eyes to provide solution in critical diagnostic cases [4]. 

ML is also known as deep learning (DL) as deep learning is a subset of machine learning, and machine learning is a subset of artificial intelligence. Deep learning is a core inner part of AI presented in 2006 by Hinton et al. [5] These terms i.e., AI, ML, and DL are used by data scientists in the multi-layered artificial neural network (ANN) for optimizing the data interpolating functions. [5] The function of ML is to handle the existing and advent of big data via various tools (e.g., Decision Tree, Naïve Bayesian Classifier, Vector Machine, Random Forest, K-Nearest Neighbor, and convolutional neural networks) along with various software. They have the ability to study relations based on the data and provide solutions. These algorithms represent Artificial neural networks (ANNs) mimicking the function of the human brain neurons. In the human brain, neurons are coupled with each other through numerous axon intersections creating charts of correlations. These links could continuously remodify to adapt new situations, relationships, and conclusions [6]. 

A huge entry of data insertion is mandatory for developing ML/DL programs. numerous features could be utilized such as clinical photographs, radiographs, text, patient symptoms, histopathological reports, and even sounds could be implemented [7]. Recent previous years, artificial intelligence in dentistry has been attracting specialties such as restorative dentistry [8], periodontics [9], oral and maxillofacial surgery [10], orthodontics [11], endodontics [12], and prosthodontics [13]. Many studies were conducted and showed encouraging results, although many programs are still in the developmental process.

Previous systematic review, Mahmood et al. compared AI-based applications for diagnosing head and neck cancer, incorporating diverse imaging modalities; histopathological and radiological feeding data [14]. Other studies utilized clinicopathologic/genomic details. They found that 69% of work were ML methods while 25% of studies were deep learning (DL) methods, and 6% of approaches had a combination of both [15, 16]. This demonstrates a growing number of studies on AI/ML in detecting head and neck cancer, employing numerous imaging modalities [14]. 

Up to the authors knowledge there is limited literature regarding utilization of AI- based program that could analyse clinical data as well as histopathological findings of oral diseases to provide a reliable differential diagnosis that help doctors during clinical and lab practice as well as in medical education. So, an urgent need for dentists to recognize the conceptualization of AI in the field of oral diagnosis and histopathological reporting and modify to the rapidly advancing healthcare protocols [17] The objective of this study was developing a software with smart algorithms contains all needed feeding data to act as AI-based program to diagnose oral diseases with accuracy. So our research question was: *Can we develop AI-based Software for accurate diagnosis of oral diseases based on clinical and histopathological data inputs that could perform accurately as expert pathologists?*

## Materials and methods

The ethical committee of faculty of dentistry, Cairo University approved this study **(**approval number 22-4-23) and in accordance with the Declaration of Helsinki and its later modifications. Consents were obtained from all cases enrolled in this research to permit the use of the clinical photos and other investigation data. This study we conducted following the CLAIM checklist guidelines.

### Study sample

The study sample included clinical images; patient symptoms, radiographic images, histopathological images and texts for the oral diseases of interest in the current study (salivary gland neoplasms, premalignant lesions, immune mediated, oral cancer, and oral reactive lesions). The inclusion criteria: all diseases of interest should be supplied with full demographic data, results of clinical investigations and complete histopathological reporting. Any case with missing data was excluded.

Total number of oral diseases enrolled in this study was 28 diseases retrieved retrospective from the archives of oral maxillofacial pathology department, Cairo University. Each disease was presented by 100 images and complemented by 400 texts (demographic data, sign, symptoms and histopathological findings) in the feeding data of the model.

Total 11,200 texts and 3000 images (*2850* images were used for training data and 100 images were used as test data to the program and 50 cases for calculating accuracy, sensitivity& specificity).

Two experts’ oral pathologists (Ph.D. holders 5 years’ experience) diagnosed the test cases using the clinical, radiographic and hematoxylin & eosin-stained slides under microscope to reach the final diagnosis with kappa agreement 0.9. The diagnostic accuracy study included nine examiners (oral pathologists, master holders with one year experience) divided into three groups to diagnose the test data. Group 1(software users): involved three different oral pathologists independently used the program (Diagnosis Oral diseases Software DODS), they utilized clinical, radiographs and histopathological images, group 2 (microscope users): involved three different oral pathologists independently used the standard microscopic examination of hematoxylin & eosin-stained tissue section slides with clinical and radiographic data. Group 3 (hybrid): involved three different oral pathologists independently used the standard microscopic examination as well as Software program to reach the final diagnosis.

All groups were blinded to test cases without previous knowledge to the final diagnosis. Inter-observer ratio was calculated for all groups. Correct diagnosis rate was calculated using the percent of correct diagnosis to each observer. The diagnostic accuracy, sensitivity and specificity were calculated by exposing the program versus the standard microscope method by oral pathologists (master holders) to 25 cases of oral squamous cell carcinoma and another 25 cases of normal oral mucosal tissue samples.

### Steps of developing diagnostic performance of the software program (DOD)

**Fig. 1:**
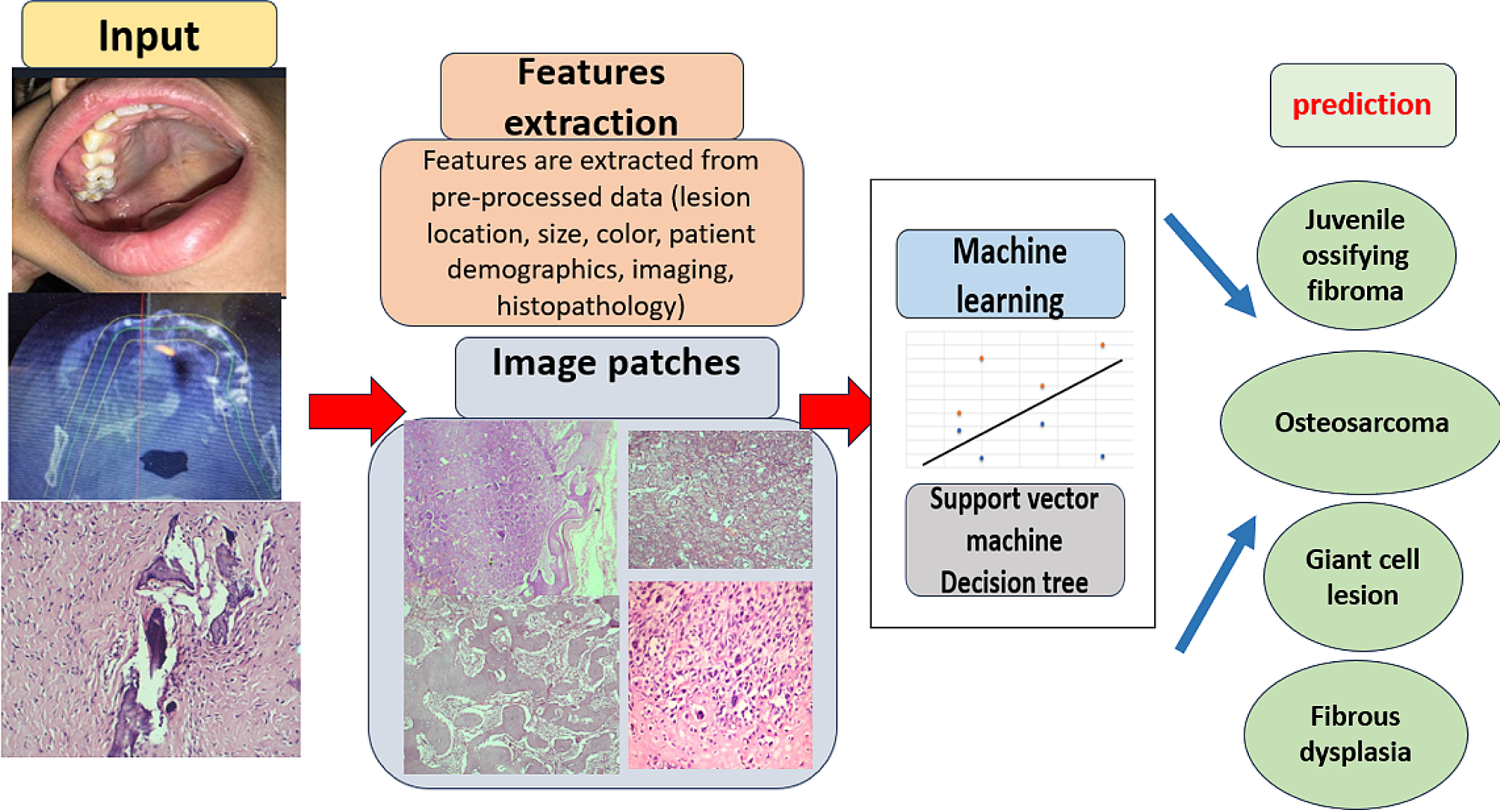
An image explaining deep learning process by supplying the program with training data to create the learning model

**Fig. 2:**
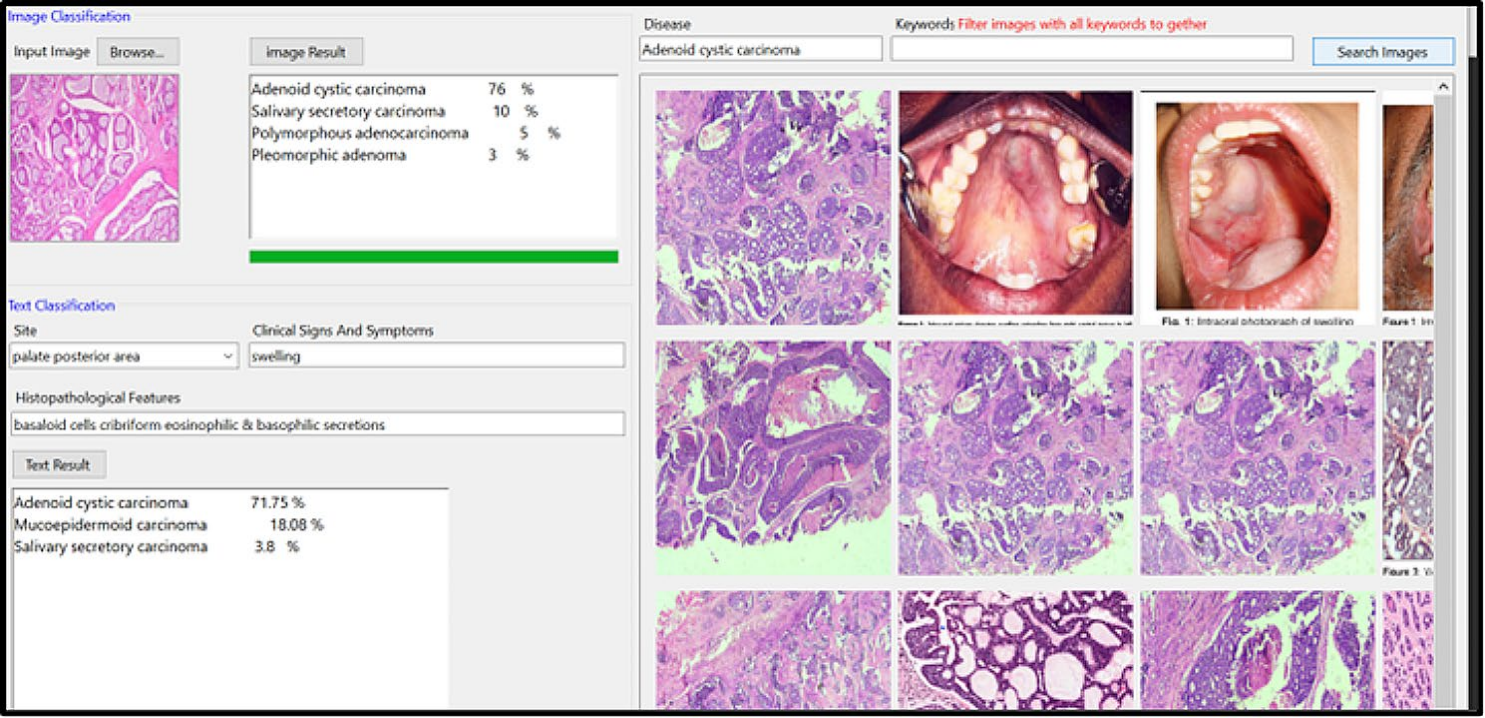
An image on computer display of the DOD program showing example of the results for the test disease. The user can browse the test photo & provide the program with key words related to site, signs, symptoms & histopathological findings of the case to be diagnosed

**Fig. 3:**
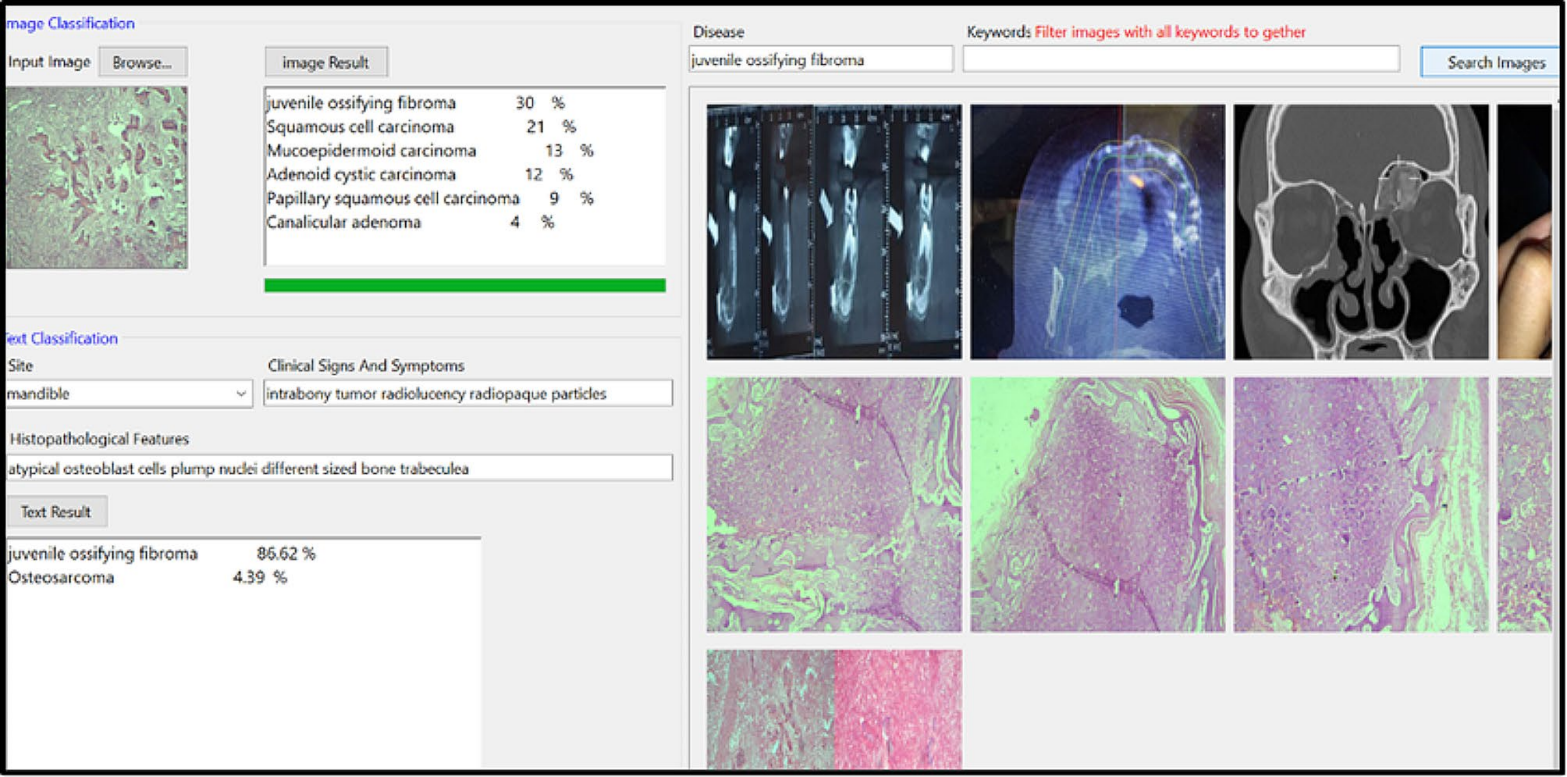
The DOD program analyze the new photo to provide users with the possible differential diagnosis with percent of diagnostic accuracy to help the doctors for further investigations and confirmation of the diagnosis

The diagnostic performance of DOD was developed using ML.Net Model Builder [18] on a CPU system equipped with an Intel Core i7 processor, Intel(R) HD Graphics and 16 gigabytes of RAM. ML.NET Classification by Binary classification algorithms and Multiclass classification algorithms. (Figures 1, 2 and 3) Training, validation, and testing procedures were executed on the hardware configuration. The chosen architecture and framework ensured seamless processing of image classification utilizing decision tree algorithm [19]and text specifications by support vector machine algorithm [20] for oral diseases of the current study.

The training and validation tasks were meticulously managed to ensure optimal learning rates that were achieved over 400 epochs. This approach allowed for the determination of the most effective parameters for creating the learning model [21]. The diagnostic achievements were analyzed for the DOD software group versus other groups by calculating the correct diagnosis rate as well as internal consistency.

The workflow how inputs were managed and preprocessing was conducted, (a) loading data: ML.NET allowed loading of data from the interpreted sources by two experts (kappa agreement 0.9) including text files, and binary files. Data is loaded into an IDataView, which is a flexible, efficient way of describing tabular data. (b) splitting data: into training, validation, and test sets to prevent the model from having prior knowledge of the unseen data. Initially, we performed random sampling to select cases for each set, ensuring that there was no overlap between them. Additionally, we implemented temporal splitting, where data from different time periods were allocated to different sets, further enhancing the independence between them. (c) data transformation: application of a series of transformations to preprocess the data. These transformations are built into a pipeline that is followed by the training algorithms. K-fold cross validation to estimate how accuracy the model will perform in practice. (d) pipelines execution: for training model and data transformation. (e) feature selection & extraction: ML.NET provided advanced preprocessing like feature selection (pick a subset of useful features) and feature extraction (synthesizing a lower set of new features) (https://learn.microsoft.com/en-us/dotnet/machine-learning/how-to-guides/prepare-data-ml-net).^(18)^

### Statistical analysis

Categorical data represented by correct diagnosis rate among the groups were presented as frequency and percentage values. Internal consistency of the results for all groups was expressed using Cronbach’s alfa. Interobserver reliability was tested using the intraclass correlation (ICC) coefficient. Accuracy was represented using the terms sensitivity, specificity, +ve predictive value, -ve predictive value, and overall accuracy. Differences between measurements were analyzed using McNemar’s test. Agreement analysis was done using Cohen’s Kappa coefficient. The difference in sensitivities and specificities was tested using chi-square test based on the method devised by Hawass [22]. Confidence intervals were calculated for binomial proportions using Clopper-Pearson’s method [23] and for other diagnostic measures using bootstrapping. The significance level was set at *p* < 0.05 within all tests. Statistical analysis was performed with R statistical analysis software version 4.3.2 for Windows [24]. 

Performance metrics.

The performance metrics are accuracy, confusion matrix, precision, recall (sensitivity), and F-scores.

Sensitivity (Recall/Hit-Rate) = True(+)ve ÷ [True(+)ve + False(-)ve]

Specificity = True(-)ve ÷ [True(-)ve + False(+)ve]

Positive predictive value (precision) = True(+)ve ÷ [True(+)ve + False(+)ve]

Negative predictive value = True(-)ve ÷ [True(-)ve + False(-)ve]

Overall accuracy (confusion matrix)= [True(+)ve + True(-)ve] ÷ All sample.

Confusion matrix is a metric model in AI assessment. It gives the matrix form of output and describes the complete performance of this model. The results of confusion matrix prediction outcomes were always in tabular representation with any one of the binary classifiers. It is used to describe the performance of the classification of an ML model with a set of test data when true values are known. Quantitatively accuracy of this confusion matrix can be calculated by using the following formula.

F1 Score is another metric of performance metrics for the evaluation of binary classification models. It makes the predictions for the positive class. It is measured based on the calculated result of precision and recall data. It is calculated by the following equation: F1 Score = 2*[precision*recall] ÷ [precision + recall].

Likelihood ratios (LR) in medical testing are used to interpret diagnostic tests. The higher the ratio, the more likely they have the disease or condition. The formula for the likelihood ratio (LR) is:

likelihood ratio = [probability a person with the condition has certain test results] ÷ [probability a person without the condition has certain test results].

## Results

The correct diagnosis rates for group 1 (DODS users), group 2 (microscopic users) and group 3 (hybrid) were 87%, 90.6, 95% respectively. (Table 1) The reliability statistical test for inter-observer value was done by calculating Cronbach’s alpha and interclass correlation coefficient. The test revealed for group 1, 2 and 3 the following values respectively 0.934, 0.712 & 0.703. All groups showed acceptable reliability. (Table 2)

**Table 1:** Showing the correct diagnosis rate between the nine examiners

Users	Total number of test cases	False diagnosis		Correct diagnosis		Correct diagnosis rate (separate/consensus)
		frequency	Percent	Frequency	percent	
DODS user 1	*N* = 100	13	13%	87	87%	87%
DODS user 2		12	12%	88	88%	
DODS user 3		14	14%	86	86%	
Microscope user 1		11	11%	89	89%	90.6%
Microscope user 2		9	9%	91	91%	
Microscope user 3		8	8%	92	92%	
Hybrid 1		6	6%	94	94%	95%
Hybrid 2		5	5%	95	95%	
Hybrid 3		4	4%	96	96%	

**Table 2:** Showing inter-observer reliability test

Groups of the study	Cronbach’s Alpha	Intraclass Correlation Coefficient						
		Intraclass Correlation(a)	95% Confidence Interval			F Test with True Value 0		
			Lower bound	Upper bound		value	df1	Df2
Group 1 (DOD Software)	0.934 For 3 items	Average measure (b) 0.934	0.908		0.953	15.040	99	198
Group 2 (Microscope)	0.712 For 3 items	Average measure 0.712	0.598		0.797	3.457	99	198
Group 3 (hybrid)	0.703 For 3 items	Average measure 0.703	0.586		0.792	3.356	99	198

a Two-way mixed effects model where people effects are random and measures effects are fixed

b Type C intraclass correlation coefficients using a consistency definition-the between-measure variance is excluded from the denominator variance

This estimate is computed assuming the interaction effect is absent, because it is not estimable otherwise

**Table 3:** Confusion matrix for master’s degree holders’ measurements

MSc PhD	*n* (%)		χ^2^	*p*-value	Cohen’s kappa (95%CI)
	Negative	Positive			
**Negative**	22 (88.00%)	3 (12.00%)	**0.20**	**0.655**	0.800 (0.629:0.971)*
**Positive**	2 (8.00%)	23 (92.00%)			

CI; Confidence Interval, *Significant (*p* < 0.05)

**Fig. 4:**
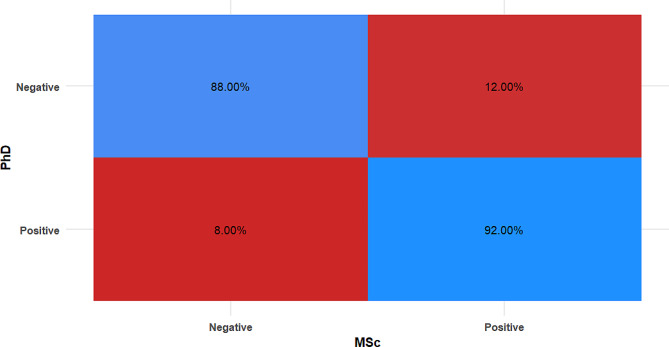
Heat map showing confusion matrix for MSc measurements

The accuracy, sensitivity, and specificity of the DODS system were respectively 82%, 84% and 80% while for group 2 examiners (master holders with 1 year experience) the corresponding values were 90%, 92% & 88%. Results of inter and intra-group comparisons for clinical scores are presented in Table 3, and Fig. 4. There were only 3 misdiagnoses made by master’s degree holders (i.e., 3 false positives and 2 false negatives). The difference between their measures and that of the gold standard (PhD holders) was not statistically significant (*p* = 0.655), and the agreement was strong and statistically significant (k = 0.800, *p* < 0.001).

**Fig. 5:**
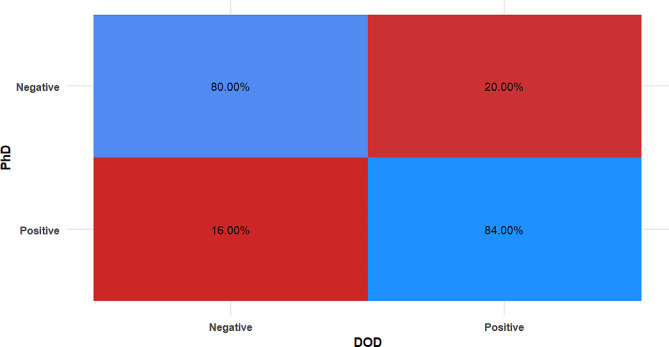
Heat map showing confusion matrix for DOD measurements

For DODS measurements (Table 4), and Fig. 5 the count of misdiagnosed cases was higher (i.e., 5 false positives and 4 false negatives) and the agreement was moderate and statistically significant (k = 0.640, *p* < 0.001), yet the difference from the gold standard was not statistically significant (*p* = 0.739).

**Table 4:** Confusion matrix for DOD measurements

DOD PhD	*n* (%)		χ^2^	p-value	Cohen’s kappa (95%CI)
	Negative	Positive			
**Negative**	20 (80.00%)	5 (20.00%)	**0.11**	**0.739**	0.640 (0.422:0.858)*
**Positive**	4 (16.00%)	21 (84.00%)			

CI; Confidence Interval, *Significant (*p* < 0.05)

**Fig. 6:**
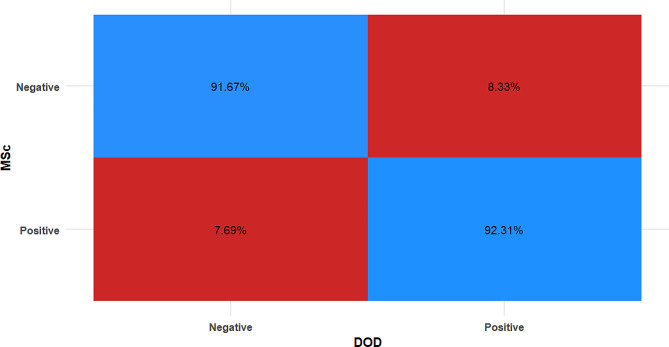
Heat map showing the agreement between tested measurements

There was a disagreement in diagnosis between master’s degree holders and DODS in 4 cases, however, the difference was not statistically significant (*p* = 1) and the agreement was strong and statistically significant (k = 0.840, *p* < 0.001). (Table 5) and Fig. 6.

**Table 5:** Difference between master’s degree holders’ measurements and DOD

DOD MSc	*n* (%)		χ^2^	p-value	Cohen’s kappa (95%CI)
	Negative	Positive			
**Negative**	22 (91.67%)	2 (8.33%)	**0.00**	**1**	0.840 (0.685:0.994)*
**Positive**	2 (7.69%)	24 (92.31%)			

CI; Confidence Interval, *Significant (*p* < 0.05)

For master’s degree holders measurements, sensitivity (true positive rate) was 92.00% (73.97%:99.02%), specificity (true negative rate) was 88.00% (95% CI; 68.78%:97.45%), positive predictive value (PPV) (the probability that a positive diagnosis is correct) was 88.46% (95% CI; 69.85%:97.55%), negative predictive value (NPV) (the probability that a negative diagnosis is correct) was 91.67% (95% CI; 73.00%:98.97%), positive likelihood ratio (LR+) (the likelihood that a positive diagnosis is correct) was 7.67 (95% CI; 2.64:22.30), negative likelihood ratio (LR-) (the likelihood that a negative diagnosis is correct) was 0.09 (95% CI; 0.02:0.35) and the overall accuracy (the probability of true diagnosis) was 90.00% (95% CI; 78.19%:96.67%). The F1 score value was 0.902 (95% CI; 0.809:0.980) (the harmonic mean of precision (PPV) and recall (sensitivity)) (i.e., as the value is close to 1 it indicates a good balance between precision and recall). (Table 6)

**Table 6:** Difference in accuracy between master’s degree holders’ measurements and DOD

Parameter	Value (95% CI)		Test statistic	*p*-value
	MSc	DOD		
**Sensitivity** ***(recall)***	92.00% (73.97%:99.02%)	84.00% (63.92%:95.46%)	**4.00**	**0.135**
**Specificity** ***(precision)***	88.00% (68.78%:97.45%)	80.00% (59.30%:93.17%)		
**PPV**	88.46% (69.85%:97.55%)	80.77% (60.65%:93.45%)		
**NPV**	91.67% (73.00%:98.97%)	83.33% (62.62%:95.26%)		
**PLR**	7.67 (2.64:22.30)	4.20 (1.88:9.37)		
**NLR**	0.09 (0.02:0.35)	0.20 (0.08:0.50)		
**Accuracy**	90.00% (78.19%:96.67%)	82.00% (68.56%:91.42%)		
**F1 score**	0.902 (0.809:0.980)	0.824 (0.703:0.927)		

CI; Confidence Interval

For DODS, sensitivity was 84.00% (95% CI; 63.92%:95.46%), specificity was 80.00% (95% CI; 59.30%:93.17%), PPV was 80.77% (95% CI; 60.65%:93.45%), NPV was 83.33% (95% CI; 62.62%:95.26%), LR + was 4.20 (95% CI; 1.88:9.37), LR- was 0.20 (95% CI; 0.08:0.50), the overall accuracy was 82.00% (95% CI; 68.56%:91.42%) and F1 score was 0.824 (95% CI; 0.703:0.927) (the value is also close to 1 that indicates a relatively good balance between precision and recall). The measurements made by master’s holders had higher sensitivity and specificity, yet the difference was not statistically significant (*p* = 0.135). (Table 6)

A representative image on computer displays of the DODS program (Fig. 2) showed an example of the results for the test disease. The user can browse the test photo & provide the program with key words related to site, signs, symptoms & histopathological findings of the case to be diagnosed. The DODS program analyzes the new photo to provide the users with the possible differential diagnosis with percent of diagnostic accuracy to help the doctors for further investigations and confirmation of the diagnosis. (Fig. 3)

## Discussion

There was a growing interest in the field of oral pathology toward utilizing artificial intelligence to provide programs that enhance the histopathology diagnostic process. Many algorithms can be used to identify patterns that could be applied to differentiate histopathological features of oral disease and provide reliable differential diagnostic list which guide less experience oral pathologists for accurate diagnosis [21]. 

The diagnostic chain of oral surgery and oral pathology to reach final diagnosis and proper treatment plan might be enhanced by machine learning algorithms like SVM, ANN, RF, and *k*-nearest neighbors, which have been scrutinized to recognize oral cysts, tumors, oral cancer, lymph node involvement and salivary gland disease. Many of these investigations revealed excellent results for ML performance. But models that assimilate more medical details about the patient are still in need to achieve accurate diagnosis [2]. 

Artificial intelligence could foster early identification of oral cancer leading hopefully to reduction of death rates and disability [25, 26]. The current study was developed to explore the power of deep learning system as a diagnostic assistance for less experienced oral pathologists and general practitioners to avoid the oversight of life-threatening conditions like oral potentially premalignant lesions and oral cancer.

The DODS is an AI-based desktop program that works on image and text classification technologies. DODS was designed to cater two main functionalities: image Classification and text Classification By utilizing decision tree and support vector machine learning algorithms to provide accurate diagnosis of oral diseases [20]. 

The presented DODS program was trained for twenty-eight oral diseases as preliminary diagnostic accuracy study; we supplied the program with 2850 images as well as 11,200 texts for training and validation of the learning model. Since the histopathological diversity of the microscopic features of oral diseases, the DODS was enhanced by bars to insert key words related to the demographic data of the test case as well as key words related to the histopathological findings in the test image.

The correct diagnosis rate of group 1 DODS was 87%, group 2 revealed accuracy of 90.6% while group 3 (hybrid) where the oral pathologists utilized both microscopic examination and images by DOD revealed significant higher diagnostic accuracy by 95%. All groups revealed acceptable interobserver reliability and interclass correlation coefficient (0.934, 0.712 & 0.702 respectively). The accuracy, sensitivity, and specificity of the DOD software system were respectively 82%, 84% and 80% while for group 2 examiners (master holders with 1 year experience) the corresponding values were 90%, 92% & 88%.These results demonstrate acceptable internal consistency among examiners in each group. Worth noting that the results of the DODS might depend to some extent on the insertion of the key words, which might differ between users, but the program was developed to provide differential diagnostic list that guide users for correct final diagnosis.

In a Japanese study, the investigators used deep learning machine (DL) program to disclose fatty degradation of parenchyma of salivary glands on CT images, which is perceptible in Sjogren’s syndrome cases. The study incorporated 500 CT images (400 images of the control and the Sjogren’s syndrome patients) were used as a feeding data while 100 images were operated as the assessment data to inspect the work of the ML system. The diagnostic performance of DL was comparable to the experienced radiologists and significantly supercilious to less experienced radiologists. They reported that the accuracy, sensitivity, and specificity of the deep learning system were respectively 96.0%, 100% and 92.0% which was equivalent to the more experienced radiologists, their values were 98.3%, 99.3% and 97.3%, while those of less experience were 83.5%, 77.9% and 89.2%.^(26)^

The deep learning system for oral diseases was previously introduced by Nayak et al. (2006) [27]. they used artificial neural network to differentiate between normal as well as premalignant tissues using laser-induced auto fluorescence. Parameters inputs like mean, spectral residual and total energy were used to teach the model. The used ANN was a multiplayer-forward type with a back-propagation algorithm for training. The results demonstrated an accuracy of 98.3%, specificity of 100%, and sensitivity of 96.5%, suggesting that this method could be useful in clinical practice [28]. 

Uthoff et al. (2017) [29] used CNN to catch precancerous and cancerous lesions from auto fluorescence images. CNN was more competent than specialist examiners in identification of lesions. They concluded that the function of the CNN model can be boosted with larger information sets. Another study by Aubreville et al. (2017) [30] utilized DL to point out oral cancer with confocal laser endomicro scopy images. This method displayed an accuracy of 88.3% with a specificity of 90%.

Shams and Htike [31] performed a comparative research to anticipate occurrence of oral cancer from oral potentially malignant lesions using deep neural networks (DNN). Achievements of DNN were examined versus to support vector machine algorithm. They found that DNN revealed superior accuracy rate of 96% contrary to the other systems.

Ozden FO [32] Developed machine learning unit based on SVM, DT, and ANNs to classify periodontal diseases based on bone loss images. Their study included 150 patients (100 as training data and 50 for testing data). DT and SVM were foremost to sort out periodontal diseases with accuracy 98% while ANN showed the least correlation between input and output variable with accuracy 46%.

Another study presented by Dank et al. (2021) [33] they developed software program based on deep neural network (DNN) to detect periodontal bone loss using periapical radiographs. Their study included 63 patients, their radiographs were used to train the program then the extent of bone loss was measured based on dental land marks on the radiographs using the DNN model. The system achieved total accuracy 89.9% which was considered a promising result with recommendation of improvement by larger data sets.

The current study revealed 87% diagnostic accuracy rate of DODS program developed by decision tree & support vector deep learning algorithm system for prediction of the possible diagnostic list enhanced by smart machine diagnosis matching percent accuracy. Its diagnostic accuracy rate was comparable to group 2 (oral pathologists master holders using microscope), while, the accuracy, sensitivity & specificity of this software was lower than those of oral pathologists (master’s degree with one year experience).

Recent systematic review by Warin, K., Suebnukarn, 2024, they screened studies conducted to investigate the use of DL in oral cancer, they found most of the studies showed relatively high accuracy, sensitivity, and specificity of DL for the diagnosis of oral cancer exceeding 80%. But due to heterogeneity in study production and delineation was high hindering proper comparisons between studies. This review found that the included studies lacked details on the annotation process, did not mention the separation of the test dataset and the proportion between training, validation, and test dataset, which resulted in a high risk of bias. Moreover, seven diagnostic researches that reported the annotation process were managed by one expert with lacking inter-annotator agreement [34]. While, in our study we enhanced the independence of our data sets and prevented data leakage as we implemented a rigorous approach during the division of data into training, validation, and test sets. Initially, we performed random sampling to select cases for each set, ensuring that there was no overlap between them. Additionally, we implemented temporal splitting, where data from different time periods were allocated to different sets, further enhancing the independence between them. The interpretation process of data was done by two experts with kappa agreement 0.9. Moreover, we assigned nine examiners of oral pathologists that were totally blind to the test data and independently diagnosed the test cases. Then, we monitored internal consistency of the results for all groups that was expressed using Cronbach’s alfa. Interobserver reliability was tested using the intraclass correlation (ICC) coefficient.

For DODS measurements, the difference from the gold standard was not statistically significant (*p* = 0.739), however, the count of misdiagnosed cases was higher (i.e., 5 false positives and 4 false negatives) and the agreement was moderate and statistically significant (k = 0.640, *p* < 0.001). It was noticed that there was a disagreement in diagnosis between master’s degree holders and DOD in 4 cases, however, the difference was not statistically significant (*p* = 1) and the agreement was strong and statistically significant (k = 0.840, *p* < 0.001). The measurements made by master’s holders had higher sensitivity and specificity, yet the difference was not statistically significant (*p* = 0.135).

Thus, the present study demonstrated that the hybrid group which used both the standard microscopic examination of hematoxylin and eosin-stained section as well as images tested by DODS revealed significant superior diagnostic accuracy rate. Also the F1 score was 0.824 which determine the harmonic mean of precision (PPV) and recall (sensitivity) indicating a good balance between precision and recall. That was relatively closer to that of the master’s degree holders where the F1 score value was 0.902. This might confirm that in the field of oral pathology, the human analytical experience is mandatory to reach accurate final diagnosis because the histopathological architecture of oral lesions always demonstrates numerous diversities of cells arrangements and different patterns. So, using deep learning machine program for histopathological examination could be useful in providing reliable differential diagnostic list that might guide the oral pathologist and less experienced clinicians for proper investigations and analysis to reach accurate final diagnosis.

The present study had certain limitations; the DODS program will need continuous deployment by adding more images to enhance the artificial intelligence power of this software as well as introduction of more oral diseases to help oral pathologists and clinicians to use the program on a wider scale. However, the appropriate feeding of input data images and selection of exact algorithms are essential for the achievement of prediction accuracy to overcome any potential biases in the AI model due to the dataset’s nature [35]. Currently, several hybrid models are available. These programs are developed for the enabling of rapid experimentation with simple, flexible, and powerful actions. It makes the segmentation of input images with trained data. Then, mix over the data images and produce excellent results with a comparison of other images via segmentation algorithms [35]. 

## Conclusions

The DODS program could be utilized as diagnostic guidance tool with high reliability. Continuous deployment of DOD software by new images of new different oral diseases is mandatory to improve the diagnostic accuracy, sensitivity, and specificity of the program. This DODS could be utilized in the field of medical education to provide students with problem solving cases to enrich their differential diagnostic skills. We believe that human-to-human communications cannot be replaced completely by computer language so the hybrid models could provide rapid, simple, flexible, and reliable actions.

## Recommendations

The DODS program diagnostic accuracy could be improved with larger data set and employment of new algorithms capable of achieving advanced deep learning correlation. In the future, we intend to provide the program with more demographic data, histopathological findings and images of different oral diseases to enhance the training process of the program for accurate reliable diagnosis and compare its performance with other AI models of same objective by collaboration of many researchers in the medical field and digital science and possibility of merging the model with a database.

## Data Availability

All data generated are included in the current manuscript.
